# UNIQUE TRANSCRIPTOME OF AGE-ASSOCIATED VISCERAL FAT-RESIDENT GAMMA DELTA T CELLS

**DOI:** 10.1093/geroni/igz038.3496

**Published:** 2019-11-08

**Authors:** Marlene E Starr

**Affiliations:** University of Kentucky, Lexington, Kentucky, United States

## Abstract

Gamma delta T cells (Tγδ) are a unique group of immune cells that perform both adaptive and innate functions. We recently identified a population of Tγδ cells which show an age-dependent expansion in the visceral fat of both mice and humans. However, little is known regarding the role of these cells in the fat. The purpose of this study was to begin delineating their role by: (1) comparing the gene expression profile of visceral fat-resident Tγδ to conventional T cells (Tconv) and to circulating Tγδ, and (2) identifying age-dependent changes in gene expression within the visceral fat-resident Tγδ population. Tγδ and Tconv were magnetically purified from blood and visceral fat of young and aged mice. Using NanoString technology, we found that overall transcriptomes of Tγδ in fat and blood were strikingly different. Transcriptomes of Tγδ and Tconv within the fat were more similar, but distinct with the former having high representation of pathways related to inflammation, cytokine and chemokine signaling, and macrophage function and the latter having high representation of pathways related to T- and B-cell function and TNF superfamily. Within the Tγδ population we identified significant (>1.8-fold change, p<0.01) age-associated differences in expression for 8 upregulated genes (C6, Cxcl13, Prg2, Il5ra, Ctla4, Marco, Ccl8, Il10) and 10 downregulated genes (Col4a1, Xcl1, Col1a1, Cfd, Col3a1, Lbp, Thbs1, Klrd1, Il6st, Ccl17). These genes will guide further research aimed at understanding the role of these cells and how their age-associated changes contribute to chronic inflammation, an underlying component of multiple age-related diseases.

